# Oscillatory Correlates of Visual Consciousness

**DOI:** 10.3389/fpsyg.2017.01147

**Published:** 2017-07-07

**Authors:** Stefano Gallotto, Alexander T. Sack, Teresa Schuhmann, Tom A. de Graaf

**Affiliations:** ^1^Department of Cognitive Neuroscience, Faculty of Psychology and Neuroscience, Maastricht UniversityMaastricht, Netherlands; ^2^Maastricht Brain Imaging CentreMaastricht, Netherlands

**Keywords:** consciousness, awareness, vision, correlates, oscillations

## Abstract

Conscious experiences are linked to activity in our brain: the neural correlates of consciousness (NCC). Empirical research on these NCCs covers a wide range of brain activity signals, measures, and methodologies. In this paper, we focus on spontaneous brain oscillations; rhythmic fluctuations of neuronal (population) activity which can be characterized by a range of parameters, such as frequency, amplitude (power), and phase. We provide an overview of oscillatory measures that appear to correlate with conscious perception. We also discuss how increasingly sophisticated techniques allow us to study the causal role of oscillatory activity in conscious perception (i.e., ‘entrainment’). This review of *oscillatory correlates of consciousness* suggests that, for example, activity in the alpha-band (7–13 Hz) may index, or even causally support, conscious perception. But such results also showcase an increasingly acknowledged difficulty in NCC research; the challenge of separating neural activity necessary for conscious experience to arise (prerequisites) from neural activity underlying the conscious experience itself (substrates) or its results (consequences).

## Introduction

In the last few decades, progress in technology and signal analysis have resulted in new neuroimaging and electrophysiology techniques, greatly enhancing the range and resolution of brain research applications. As such, our understanding of the brain has proceeded at a staggering pace. Naturally, these techniques have been tried on the oldest problem of all: the nature of consciousness.

‘Consciousness’ can be defined in many ways (for our own taxonomy, see de Graaf et al., 2012; de Graaf and Sack, 2014). Generally, it is useful to separate minimally two concepts of consciousness. ‘State consciousness’ determines consciousness on a global level, for example distinguishing the extent of consciousness in coma, wakefulness, or anesthesia (e.g., Koch, 2004; Laureys and Tononi, 2010). ‘Content consciousness’ refers to moment-by-moment experiences of a conscious being, such as the experience of seeing blue, hearing a trumpet, or the famous ‘what-it-is-like’ to momentarily be a bat (Nagel, 1974). In this article, we focus on content consciousness, specifically in the visual modality.

The neural correlates of consciousness (NCCs) have been defined as the minimal set of neuronal mechanisms that are jointly sufficient for a conscious experience (Crick and Koch, 1990b).

To study NCCs, one generally tries to induce minimally two different conscious experiences using ‘consciousness paradigms’ [e.g., illusions, multistable and ON–OFF paradigms (Kim and Blake, 2005; de Graaf and Sack, 2014)], to then measure and compare brain activity in both (with neuroimaging techniques). This basic approach has been referred to as ‘contrastive analysis’ (Baars, 1989; Aru et al., 2012). It can help reveal endogenous neural mechanisms underlying conscious perception, particularly if the physical stimuli remain identical in both conscious states. For example, when a low-intensity visual stimulus is repeatedly presented at perception threshold, the participant consciously perceives it on some but not on all trials. Thus, under identical stimulation conditions, this creates two types of trials: trials with conscious perception (ON) and trials without conscious perception (OFF) (de Graaf and Sack, 2014).

Different neuroimaging techniques can compare brain activity in both types of trials, such as functional magnetic resonance imaging (fMRI), magneto-/electroencephalography (M/EEG), electrocorticography (ECoG), or positron emission tomography (PET). Each has distinct advantages and applications, but here we focus on M/EEG, which can detect rhythmic fluctuations of brain activity, i.e., oscillations, with high temporal resolution. This is valuable as there is increasing evidence that oscillatory signatures may index conscious perception (e.g., Hanslmayr et al., 2007; Romei et al., 2008b; Busch et al., 2009; Lange et al., 2013). We here review such evidence, organized by frequency-band. For some of the oscillatory correlates of consciousness, recent studies investigated their causal contribution to conscious perception. By using brain stimulation techniques or rhythmic sensory stimulation, fascinating new ‘entrainment’ approaches allow the experimenter to control oscillatory activity to evaluate its causal role in conscious perception. From this overview, we address the question; what are the oscillatory correlates of consciousness?

In addressing this question, this review has three goals. Firstly, it is meant to be instructive. We provide a basic overview of oscillations and how to measure them, paradigms used to identify, isolate, and study consciousness, and results: oscillatory measures reported to correlate with (visual) consciousness using such approaches. Secondly, we draw attention to the recent applications of entrainment to study the causal role of these oscillatory measures. Thirdly, we use the reviewed findings to illuminate an old problem: how to determine the functional role of such mechanisms? We have previously discussed how NCC, of any type or form, can factually be three sorts of processes: neural prerequisites, neural substrates, and neural consequences of a conscious experience (de Graaf et al., 2012; de Graaf and Sack, 2014). Interpreting oscillatory correlates of consciousness in this framework may provide new insights, and should be kept in mind when designing and interpreting future studies.

## How to Study Oscillatory Correlates of Consciousness?

### Consciousness Paradigms

**FIGURE 1:**
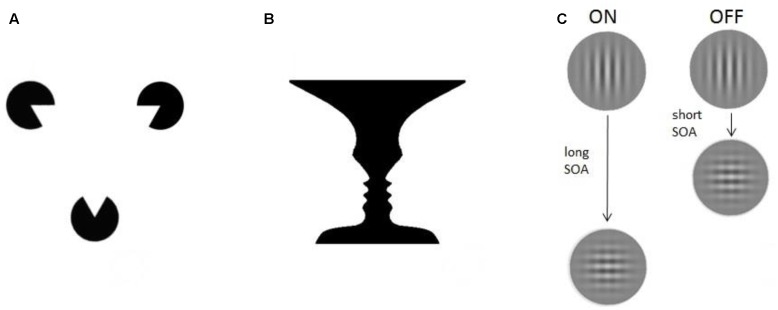
Experimental paradigms. Three different examples of consciousness paradigms: **(A)** *Illusions.* The Kanizsa triangle consists of three spherical figures, each of which misses a triangular portion (pac-men). When placed in a proper configuration the figures induce an illusory percept of triangle-contours (Kanizsa, 1976). **(B)** *Multistable paradigms.* The vase/faces figure provides a well-known demonstration of bistable perception: the same visual stimulus can alternately induce the perception of either a vase or two faces (Rubin, 1915). **(C)** *ON–OFF paradigms.* The visual masking paradigm, for example, uses two stimuli presented in spatiotemporal proximity. Depending on the time between them (stimulus onset asynchrony, SOA) the participant is conscious (ON) or not conscious (OFF) of the vertical grating.

Generally speaking, consciousness paradigms share the ability to induce at least two different conscious percepts of a physically identical stimulus (Logothetis, 1998; Blake and Logothetis, 2002). We previously grouped them into three different categories: illusions, multistable paradigms, and ON–OFF paradigms (de Graaf and Sack, 2014, 2015), illustrated in **Figure 1**.

*Illusions* are conscious percepts that are created endogenously, in absence of sensory information from the physical environment usually causing the conscious percept now observed (i.e., in other situations or in other observers). A famous example is the Kanizsa triangle (Kanizsa, 1976): one perceives triangle-contours, even though lines delineating the sides of the triangle – which usually cause the conscious triangle percept – are missing from the image. Illusions can be useful to study consciousness, since brain activity correlated to their perception reflects ‘constructive’ processes of conscious vision (Goebel et al., 1998). A different approach involves afterimages: a percept remains present in visual experience even though the stimulus that evoked it has been removed (Zaidi et al., 2012). Hallucinations, lastly, do not involve any input and might be classified as illusions as well. They are typically present in pathologies as schizophrenia, in which the patient can experience different percepts (e.g., auditory, olfactory) in the total absence of external stimulation. But in fact, many of us may perceive hallucinatory illusions if we are deprived of sensory inputs altogether (Vosburg et al., 1960).

There are other examples of illusions in the absence of sensory stimuli, from less controlled and more complex [e.g., phantom pain (Blakeslee and Ramachandran, 1998), or illusory percepts in a scotoma] to fully controlled (e.g., magnetic pulse-induced ‘phosphenes’; illusory visual experiences induced without visual stimulation (de Graaf et al., 2014). Goebel et al. (1998) provided a compelling demonstration of how to use illusions to study conscious perception. They used static visual stimuli to induce illusory contours that appeared to move (the illusory motion quartet), and mapped correlating brain activity with fMRI. By separating the features of the visual inputs (static) from the features of the illusory percept (motion), the activity observed in the human motion areas could only be attributed to the endogenous construction of conscious motion perception.

*Multistable paradigms* notably include the well-researched paradigms of binocular rivalry (Fox, 1991; Blake, 2001), and ambiguous figures, such as the famous Necker cube or Rubin’s vase/faces (see **Figure 1**). In the *binocular rivalry* paradigm, two different images are presented, each to one eye, at corresponding retinal locations. They need to be sufficiently different from each other, so that binocular fusion is impossible. As a result, the conscious percept of the observer keeps changing, even though stimulation never changes. Similarly, an observer will always experience only one conscious percept at a time when presented with a constant *ambiguous figure*, such as Rubin’s vase/faces (**Figure 1B**), where the observer either experiences the vase, or the face, but never both simultaneously. In binocular rivalry, and here, comparing brain activity during both possible percepts can be very useful to find NCC’s, because there is a change in consciousness unaccompanied by a change in external inputs. Any change in brain activity, occurring together with the change in consciousness, can be interpreted as underlying conscious processing of whichever percept is now reported. These correlates of conscious percepts are then not confounded by several unconscious perceptual processes that normally result from changes in inputs.

However, in those multistable paradigms, a condition is defined by the participant’s report of their conscious percept. Participants mainly signal their experience by button presses. Practically, this creates problems in neuroimaging, since brain activity correlated to such percept switches (Lumer et al., 1998; Tong and Engel, 2001) is contaminated with task performance (Knapen et al., 2011). With M/EEG, the variability in response times creates additional difficulty (Strüber et al., 2000; Strüber and Herrmann, 2002), so it is promising that new and temporally accurate measures of percept switch timing are being explored (e.g., ocular reflexes; Frässle et al., 2014).

The high temporal resolution of M/EEG makes these techniques particularly well-suited for a third class of consciousness paradigms: *ON–OFF paradigms*. ON–OFF tasks have two conscious states: ‘stimulus perceived’ (ON) and ‘stimulus not perceived’ (OFF), i.e., conscious vision present vs. not present. The implementations of this basic principle come in many forms, such as visual masking (Breitmeyer and Ogmen, 2006) or transcranial magnetic stimulation (TMS) (Taylor et al., 2010). Generally, brain activity is simply contrasted between the ON and the OFF condition. Hemodynamic imaging allows us to study consciousness using ‘weak ON–OFF tasks’ in which *small stimulus parameter adjustments* cause stimuli to be always perceived or never perceived – enabling the implementation of experimental blocks of ON and OFF trials (e.g., word masking in Dehaene et al., 2001). But M/EEG can employ ‘strong ON–OFF tasks’ in which the *exact same stimuli* are used in all trials. In this case, brain activity highlighted by contrastive analysis is strictly related to endogenous processes differentiating stimulus perceived (ON) from not perceived (OFF) conditions, since the input does not change at all. With strong ON–OFF tasks we can therefore isolate and compare even more precisely the activity related to the two conditions. As per our earlier example; the simplest form of this is visual stimuli presented at perception threshold, causing detection (ON) on half of all trials, and failure to detect (OFF) on the other half of trials.

Thus, illusions, multistable, and ON–OFF paradigms, are all suitable for brain imaging experiments employing contrastive analysis. Yet they also share a conceptual difficulty which should be noted. In the example of a stimulus detection task, ON trials can engage a neural mechanism ‘N,’ which is not activated in OFF trials. ‘N’ is therefore an empirical correlate of consciousness. But which level of processing is ‘N’ involved in? A conscious percept finally arises from a cascade of processing, much of which is unconscious and which can likely be segmented into many steps and stages depending on context and framework. Thus, the exact *role* of ‘N’ can usually not be determined from a single experiment. We return to this issue in Section “Functional Roles of Oscillatory NCCs”.

### Oscillations

To continue with the example of a detection task, once ON and OFF trials have been *post hoc* labeled based on participant responses, oscillatory activity can be contrasted between both conditions. Though, we will discuss primarily oscillatory activity as measured with non-invasive neuroimaging methods such as M/EEG, much will apply to oscillatory signals measured more invasively in humans (e.g., ECoG) or oscillatory signals from smaller populations (e.g., local field potentials). So what is ‘oscillatory activity’? A single oscillating signal can be characterized by three parameters: frequency, amplitude, and phase.

**FIGURE 2:**
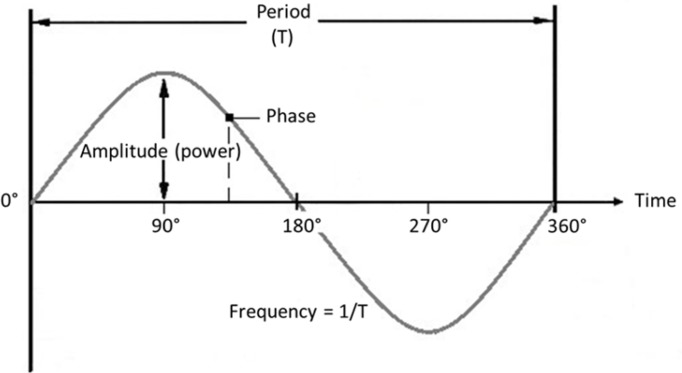
Oscillation parameters. Frequency: Number of cycles per unit of time (s). Amplitude: Strength of the signal (size of deflections from the mean). Phase: Momentary position on the cycle at a certain point in time. Period: Time duration of one cycle.

The rhythmic fluctuations in M/EEG signals primarily reflect rhythmic synchronous firing of populations of pyramidal neurons [i.e., excitatory and inhibitory postsynaptic action potentials (Creutzfeldt et al., 1966; Proudfoot et al., 2014)]. The strength of the signal, which translates to the *amplitude* (directly related to ‘power’) of an oscillation, depends on the absolute number of firing pyramidal cells, how often they fire, and to what extent they fire synchronously. This synchronization is mainly guided by interneurons which, discharging together, generate perisomatic inhibitory postsynaptic potentials (Bartos et al., 2007). The rhythmic nature of individual neuronal firing bursts results in population-level activity that follows a sinusoidal pattern, with alternating high and low levels of activity. At any point in time, where (or rather when) the signal finds itself on this repeating sinusoidal activity cycle is defined as its *phase*. How often the activity cycle goes up and down in a certain unit of time (generally seconds) is defined as the signal’s *frequency*, see **Figure 2** for a visualization.

When looking at an oscillating signal across repeated trials, one could analyze how similar the phase is across trials with reference to a particular time-locked event; called *phase-locking* (Tallon-Baudry et al., 1996). When looking at brain systems, one could also evaluate phase-locking between two nodes of a brain network, evaluating how consistent the phase relationship is between two oscillating signals when a particular event occurs. Or, more generally, and independently of certain time-locking occurrences, how consistent the phase relationship is between two ongoing signals from two brain regions, in which case one is quantifying *phase coherence* (Srinivasan et al., 1999). Such measures likely reflect functional connectivity between regions, and while there is a whole range of more advanced analyses one might consider in such contexts, for instance to evaluate directed connectivity (which region drives activity in the other?), these are beyond the scope of this review (see Bastos and Schoffelen, 2015 for a recent review of advanced analyses).

**FIGURE 3:**
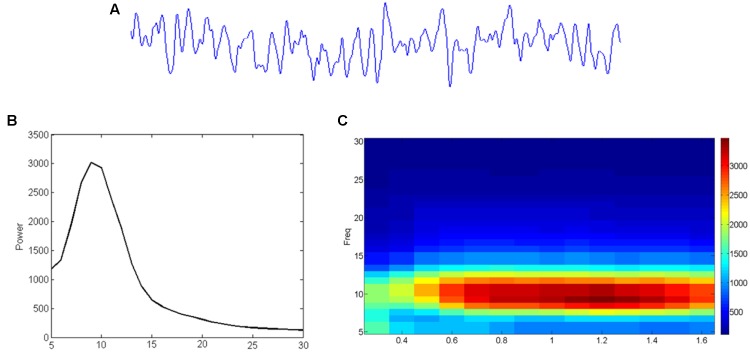
From EEG time-signal **(A)** to a frequency **(B)** or even time-frequency **(C)** representation. **(A)** The time signal reflects how much signal (voltage) is picked up at an electrode/sensor at subsequent sample points. **(B)** A Fourier analysis can reveal to what extent (power; vertical axis) sinusoids in different frequencies (horizontal axis) contribute to it. **(C)** Similar analysis can reveal the development (time; horizontal axis) of such frequency (vertical axis) contributions (color-coding).

Data obtained with M/EEG measurements reflect a combination of noise and signals, which can be analyzed in different ways. As shown in **Figure 3**, one can extract the contribution of oscillatory signals in different frequencies (**Figure 3B**) to the original (preprocessed) data (**Figure 3A**), or visualize how these contributions change over time (**Figure 3C**). Oscillatory brain activity itself can fluctuate over time, and different networks in the brain are characterized by different frequencies (Keitel and Gross, 2016). For instance, occipital and parietal brain areas are mostly characterized by alpha activity, and sensory areas by alpha as well as beta activities (Pfurtscheller et al., 1996; Hari and Salmelin, 1997; Hillebrand et al., 2012). Furthermore, it has been suggested that activity within a given brain network may reflect a unitary sampling rhythm that is different between distinct networks (Canolty and Knight, 2010). For example, while small local networks usually operate in higher frequencies, larger distributed networks may employ slower fluctuations (Draguhn and Buzsaki, 2004). In line with this idea, theta/alpha-band oscillations (4–13 Hz) have been related to long-range communication, but beta/gamma-band oscillations (20–100 Hz) to short-range signaling (von Stein et al., 2000).

Moreover, certain brain systems may inherently prefer different frequency-bands, referred to as their ‘normal frequencies’ (Niedermeyer, 1999). In fact, different brain systems show particularly strong responses in different frequency-bands, measured with EEG, in response to single magnetic pulses (TMS), with occipital cortex presenting a stronger response to alpha-band oscillations, parietal cortex to beta-band oscillations and frontal regions to fast beta and gamma oscillations (Rosanova et al., 2009). But it has also been suggested that brain networks might be flexible enough to employ different frequencies depending on sensory modality, task demands or parameters (VanRullen, 2016). In sum, the engagement of oscillatory mechanisms in distinct frequency bands across regions, tasks, and brain states, remains a topic of intense investigation.

This completes our introductions into consciousness paradigms, oscillation signals, and (analysis of) oscillatory brain mechanisms. In what follows, we review current evidence for oscillatory mechanisms that correlate with conscious (visual) perception.

## Oscillatory Correlates of Consciousness

We have seen that there is a range of paradigms to study oscillatory NCCs, and a range of oscillatory parameters to evaluate. In this section, we will review key findings on oscillatory correlates of consciousness, grouped by frequency-band. Lower frequencies (delta, theta) do not have a dedicated section because we are not aware of much evidence supporting their role in conscious vision *per se*.

### Gamma Frequency

Experimental evidence for a relationship between gamma-band (high-frequency: ∼30–100 Hz) activity and conscious perception was highly influential, helping to reinvigorate the scientific study of consciousness when Crick and Koch (1990a) summarized it in the ‘40 Hz hypothesis.’ This hypothesis proposed that distributed neuronal activity is ‘bound’ through synchronization of oscillations, and that such synchronized activity specifically in the gamma-band is a neural correlate of conscious perception. Engel and Singer (2001) noted that binding by synchrony is implicated in several major processes related to conscious perception; arousal (Munk et al., 1996), segmentation (Engel et al., 1991), selection (Fries et al., 1997), and working memory (Tallon-Baudry et al., 1998). In cats, global features of visual stimuli (i.e., coherency of motion) produced gamma synchronization in the visual cortex (40–60 Hz) (Gray et al., 1989). Moreover, gamma-band synchrony directly indexed which of two incompatible images was perceived by a cat in a binocular rivalry implementation (Fries et al., 1997). In macaques, local field potential (LFP) fluctuations in the gamma range were recently correlated to phenomenal perception, higher up in the visual hierarchy in lateral prefrontal cortex (Panagiotaropoulos et al., 2012).

In humans, M/EEG studies showed that synchronization between large populations of neurons in anterior and posterior brain areas correlates to conscious vision (Srinivasan et al., 1999), occurring at global rather than local level as, for instance, it happens during the encoding of an external stimulus from a sensory area (Ward, 2003). Words that are consciously perceived, as compared to words not perceived, lead to a transient distributed gamma synchronization response, phase-locked both across and within hemispheres (Melloni et al., 2007). Furthermore, long-distance gamma synchronization appears only when perceptual objects are perceived as coherent conscious percepts (i.e., faces) as opposed to meaningless shapes (Rodriguez and George, 1999; Doesburg et al., 2005).

Gamma-band activity and consciousness have been investigated extensively, but not exclusively, in the visual domain. Gamma synchronization also correlates with conscious perception of non-visual stimuli. For example in the auditory system oscillatory activity near 40 Hz is not only related to the sensory but also to the cognitive processing of auditory clicks stimuli (Joliot et al., 1994). Furthermore, it has been related also to multimodal perception (Senkowski et al., 2008). For example, a recent study used a paradigm consisting of visual and auditory stimuli and showed that gamma power correlates with audiovisual perception (Balz et al., 2016). It can also have a functional role in the binding of distributed neural activities in olfactory consciousness (Mori et al., 2013). Lastly, tactile stimulation of one hand increases gamma-band coherence in the contralateral primary somatosensory cortex only when the stimulus is consciously perceived (Meador et al., 2002) as well as when tactile stimuli are associated to visual stimuli, showing contralateral enhancement of gamma-band activity in occipital cortex (Lange et al., 2011).

In spite of these examples, gamma oscillatory activity as a ‘signature’ of consciousness continues to be debated. Gamma synchronization may also be induced by processes such as attention (Fries et al., 1997), which should be separated from conscious perception whenever possible (Koch and Tsuchiya, 2007). In this context, Wyart and Tallon-Baudry (2008) have suggested that attention is more associated with high gamma frequency, whereas conscious perception is more associated with mid-range gamma synchronization. Yet, there is also evidence that gamma-band activity does not solely appear when consciousness is present but can also persist or even increase during anesthesia (Vanderwolf, 2000; Imas et al., 2005; Murphy et al., 2011) or seizures (Pockett and Holmes, 2009), brain states that are clearly not characterized by consciousness. It may therefore be that although gamma-band activity is present in many different conscious states, it is not exclusive to them (Hermes et al., 2015) and not sufficient to allow consciousness (Luo et al., 2009). Of course different measures of gamma-band activity have been considered in the past, from local gamma power to distributed gamma coherence, and moreover in and across different brain systems, so the picture remains incomplete.

### Beta Frequency

One example of an ON–OFF paradigm is the ‘attentional blink’ paradigm, in which a rapid stream of visual stimuli is presented at fixation (rapid serial visual presentation, RSVP, task). Participants are given two targets (i.e., specific letters) to watch out for, and press a button whenever they see either one of them. The attentional blink phenomenon is the observation that participants are more likely to miss a target, if it follows a preceding target in a particular temporal window [target 1 to target 2 onset asynchrony (stimulus onset asynchrony, SOA) of around 200–500 ms; Shapiro et al., 1997]. The ‘weak’ version of this paradigm uses two different SOAs, one leads to stimulus perception (ON) and one does not (OFF). When only one SOA is used, for which target 2 is sometimes detected and other times not, this paradigm becomes a ‘strong’ ON–OFF paradigm. Gross et al. (2004) measured MEG during such an implementation. They found increased power in the low beta-band during the entire stream of stimuli when targets were detected (ON) compared to when they were not (OFF). Furthermore, they found stronger beta synchronization in a network dominated by right inferior parietal and left prefrontal regions, in ON trials.

The enhancement of beta synchronization might reflect a general state of increased sensitivity to behaviorally relevant stimuli, which could explain better target detection performance. Gaillard et al. (2009) presented masked words at threshold contrast, in an intracranial EEG study. In detected versus non-detected trials, there was stronger beta synchronization between long-distance regions, especially during the late phase of the conscious access, whereas this coherence was suppressed when the same stimulus does not become conscious. Interestingly, in both studies the synchronized activity appears not only in posterior regions, but spreads in a broader network that involves also frontal areas.

The relationship between beta oscillations and visual consciousness is not yet fully clear. For instance, one recent study with invasive recordings in the macaque, showed that the power of beta oscillations in lateral prefrontal cortex is not modulated by conscious versus unconscious stimulus processing (Panagiotaropoulos et al., 2013). But here again, we should keep in mind that local oscillatory synchronization, i.e., local oscillatory power, may reflect at least partially non-overlapping brain processes as compared to measures of phase coherence. Synchronization across brain regions is not the same as synchronization within brain regions.

## Alpha-Band Activity

Alpha oscillations have been extensively researched in relation to conscious (visual) perception. The alpha rhythm (7–13 Hz) is strongly linked to posterior areas of the brain, and has been associated to input regulation (Lorincz et al., 2009) as well as attention (Worden et al., 2000; Sauseng et al., 2005; Kelly et al., 2006; Thut et al., 2006; Marshall et al., 2015). When our brain is not engaged in a particular task, oscillations with alpha rhythm are more prominent and easy to detect, leading to the notion that alpha is an ‘idling’ rhythm, the activity of the brain at rest (Pfurtscheller et al., 1996). For instance, simply closing the eyes strongly enhances alpha power (Berger, 1929). At the same time, a large body of research has led to several sophisticated theories on exactly which role alpha activity plays in attention, perception, and awareness. Below, we discuss in turn several parameters of alpha activity and how they have been studied using versions of consciousness paradigms.

### Alpha Power

Since ongoing alpha power does not stay at a constant level but fluctuates over time (Lopes da Silva, 1991), alpha power fluctuations have been studied in relation to fluctuations in visual target detection. For instance, across participants, Hanslmayr et al. (2005) showed that lower performance in a visual perception task, in which participants discriminated different letters, correlated to higher parieto-occipital alpha amplitudes. Also within participants, the higher pre-stimulus alpha power activity, the less likely it is that a stimulus is detected. This probability of detection can be predicted by the amount of pre-stimulus alpha power trial-by-trial (Ergenoglu et al., 2004), particularly from alpha signals originating in the parieto-occipital sulcus (van Dijk et al., 2008). One interpretation of these results suggests that alpha power indexes a state of excitability (Klimesch et al., 2007). Indeed, Lange et al. (2013) cleverly used so-called ‘double flash illusion’ and ‘fusion effect’ paradigms to distinguish whether reduced alpha power increases the accuracy of visual processing (correctly reporting the occurrence of either one or two stimuli) or rather increases visual excitability (reporting two stimuli irrespectively of the correct answer). Their findings supported the latter hypothesis.

It has been suggested that a more direct measure of visual cortex excitability can be derived from phosphene perception. Phosphenes are fleeting conscious visual experiences, elicited experimentally through direct stimulation of visual cortex (Marg and Rudiak, 1994). For instance, TMS can be used to non-invasively excite neurons in occipital cortex, which in many participants results in phosphene perception if the stimulation intensity is sufficient. Different levels of excitability can be assessed directly by evaluating the stimulation intensity required to elicit phosphenes (phosphene threshold), or the proportion of trials that result in phosphene perception at some fixed level of stimulation intensity. A lower phosphene threshold, or higher proportion of phosphene perception at fixed TMS intensity, indicates higher visual excitability. Measuring alpha power with EEG, and visual excitability with TMS, alpha power has been related to excitability (with higher alpha power indicating lower excitability) across (Romei et al., 2008b) and within (at trial-by-trial level) participants (Romei et al., 2008a).

In sum, converging evidence suggests that the power of alpha oscillations around stimulus (or TMS pulse) onset co-determines whether that stimulus reaches conscious perception.

### Alpha Phase

Inherently, oscillatory phase fluctuates more quickly than power. In the case of alpha-band oscillations, several studies have correlated visual detection performance to the phase of naturally occurring alpha oscillations at the moment of target presentation. Busch et al. (2009) showed that the threshold to detect light flashes covaries over time with alpha phase, suggesting that alpha phase might shape our perception by determining whether or not a visual stimulus is selected for awareness. Similarly, Mathewson et al. (2009) revealed that metacontrast-masked visual targets are more likely to be detected if targets are presented at the peak, as opposed to the trough, of ongoing alpha oscillations measured with EEG. Interestingly, they found that alpha phase predicted detection performance only when alpha amplitude was high. Thus, oscillatory phase and amplitude, though different measures, may be challenging to evaluate separately.

It is possible that, as we saw above for alpha power, also alpha phase directly reflects visual excitability. Once again, TMS-elicited phosphene perception has been used as a probe for occipital excitability. And indeed, phosphene perception, and thus visual excitability, depends on the phase of ongoing alpha oscillations (Dugué et al., 2011). At the same time, it has been suggested that alpha oscillations represent the time frames of perception (VanRullen, 2016): short visual ‘snapshots’ of the world are represented by single cycles of the alpha oscillation. This hypothesis has been supported by studies showing that two visual stimuli presented in a short period can be detected as one, or two, depending on the precise frequency of alpha oscillations in individual observers. The shorter the cycle is [higher individual alpha frequency (IAF)], the higher the temporal resolution of perception will be, and thus the more likely it will be that an observer can correctly detect the presentation of two separate stimuli over time (Samaha and Postle, 2015), independently of the amplitude of alpha-band activity (Milton and Pleydell-Pearce, 2016).

There is further evidence for a functional role of alpha phase in the context of conscious vision, stemming from a different category of studies to which we now turn.

## Is Oscillatory Activity Causally Involved in Visual Consciousness? Entrainment Approaches

The studies discussed so far have utilized a correlational approach, generally contrasting passively measured brain activity in trials in which a stimulus was perceived with trials in which a stimulus was not perceived. Such studies have clearly shown that oscillatory activity, namely power, phase, and coherence in distinct frequency bands, can be related to conscious vision. They do not clarify, however, whether such electrophysiological processes play a causal role in perception and awareness, or are epiphenomenal consequences of other brain mechanisms that underlie conscious perception. To evaluate the causal role of oscillations, one should find a way to manipulate oscillatory parameters externally, bringing neuronal oscillations under experimental control. This general approach is called ‘entrainment’ (Thut et al., 2011a; Herrmann et al., 2016), and can be achieved in different ways. These include rhythmic sensory stimulation and brain stimulation. Here, we briefly review evidence that these approaches can indeed affect behavioral performance and neuronal oscillations, followed by an overview of which oscillations appear to be causally relevant for conscious vision.

### Entraining Behavior

A participant presented with a stream of auditory stimuli, in a constant rhythm, can predict when an upcoming stimulus will appear. This phenomenon may be related to ‘*sensory entrainment’*; the alignment of a sensory system to the rhythm of sensory stimulation (Sameiro-Barbosa and Geiser, 2016). To test whether the sensory system of the participant is aligned with an external stimulation, researcher measure task performances as, for example, reaction time or detection accuracy. When the synchronization to the rhythm of presentation occurs, the response to an upcoming external stimulus is typically faster (i.e., lower reaction time), compared to when the entrainment is not present.

Rimmele et al. (2011) used auditory stimuli in order to test whether spatial and temporal expectations may change task performance. They used four different conditions (temporal expectation, spatial expectation, temporal and spatial expectation, and no expectation) and showed enhanced target detection and faster reaction time only in the condition of stimuli presented with temporal regularity. Furthermore, entrainment may lead to a more accurate performance. Facilitated performance has been shown in discriminating the intensity of a tone (Jones et al., 2002), as well as its duration (McAuley and Jones, 2003). In the visual domain, when gabor patches were presented within a stream of stimuli with fixed SOA, they were discriminated better compared to when SOAs in the stream were jittered (Rohenkohl et al., 2012). The same results have been shown by Marchant and Driver (2013) who, using auditory (tones) and visual (red annuli) stimuli, showed faster reaction times and improved visual sensitivity when they were presented in a isochronous (with temporal regularity) condition compared to when they were presented randomly.

### Entraining Oscillations

An important question is whether this temporal alignment (i.e., synchronization) occurs not only at behavioral level, but also between intrinsic neural activity and the rhythm of the external stimulation. Oscillatory brain activity can be entrained by stimuli of different nature (e.g., visual, auditory, tactile) which may lead to synchronization of neural activity in visual (Mathewson et al., 2012; de Graaf et al., 2013), auditory (Luo and Poeppel, 2007; Besle et al., 2011; Nozaradan et al., 2016), or somatosensory (Langdon et al., 2011; Ross et al., 2013; Ruzzoli and Soto-Faraco, 2014) brain areas. Oscillations in different frequency bands can be synchronized to external stimuli depending on the rhythm of stimulation (Lakatos et al., 2008, 2013). Rhythmic auditory stimulation, for example, can modulate neural activity in high frequency bands as beta and gamma (Snyder and Large, 2005; Fujioka et al., 2012) and even more robustly in low frequencies as delta and theta (Kayser et al., 2009; Howard and Poeppel, 2012; Ding et al., 2014).

One shortcoming of sensory entrainment is that it can be difficult to localize the brain mechanisms underlying its effects. After all, the rhythmic sensory stimuli are processed throughout a sensory system, making it difficult to evaluate the causal role of oscillations in a specific brain area of interest. Fortunately, it is also possible to entrain neuronal oscillations locally, by *directly* stimulating a brain region with a particular frequency. *Non-invasive* brain stimulation (NIBS) has been applied to study the causal contribution of brain areas to a wide variety of processes (including conscious vision, see for review: de Graaf and Sack, 2014), and recently also brain oscillations.

Transcranial magnetic stimulation and transcranial alternating current stimulation (tACS) are NIBS techniques used to entrain neuronal oscillations (Antal et al., 2008; Thut et al., 2011b). Single TMS pulses have been shown to affect oscillatory mechanisms in distinct frequency bands depending on the site of stimulation (Rosanova et al., 2009). When multiple TMS pulses are applied in a certain frequency (e.g., 10 Hz), this likely causes a resetting of the phase of oscillatory neural activity followed by amplification of local oscillatory power in that same frequency range (Thut et al., 2011b).

Transcranial alternating current stimulation uses a low-intensity alternating current (i.e., it changes direction periodically) which can affect the membrane potential. Thereby it can interact with cortical excitability, allowing the modulation of spontaneous brain activity in specific frequencies (Antal et al., 2008; Chaieb et al., 2011; Wach et al., 2013). Zaehle et al. (2010) showed that when tACS is applied at IAF, its effects last beyond the stimulation, resulting in enhanced alpha power as measured by EEG after versus before tACS. Neuling et al. (2013) suggest that the after-effect can last up to 30 min, but emerges only when tACS amplitude is greater than the endogenous IAF power. Also using an online paradigm (i.e., the stimulation is applied while EEG records neural activity), Helfrich et al. (2014) could show that oscillatory entrainment at 10 Hz in parieto-occipital areas increases alpha power. However, it appears relevant that tACS is continuous. Strüber et al. (2015) used a short intermittent protocol composed of 1.5 s of resting EEG and 1 s of tACS stimulation, showing that such short stimulation bursts did not cause entrainment.

Despite the substantial number of studies reporting entrainment, the mechanisms underlying the effect of tACS is still not completely clear. For example, the effects may depend on brain state during stimulation, such as having eyes open or closed (Ruhnau et al., 2016). Furthermore, Vossen et al. (2015) replicated with EEG that alpha frequency tACS increased power in the alpha band (for repeated 8 s but not 3 s bursts of tACS). However, these after-effects of tACS were observed independently of whether sequential bursts of tACS were in phase or not. Also, EEG alpha oscillations immediately following tACS bursts did not phase-align with the preceding tACS burst. Lastly, the peak frequency in the alpha band after tACS did not correspond well with the exact tACS frequency, rather reflecting IAF. These results led the authors to propose a different hypothesis regarding the after-effects of tACS stimulation; reflecting synaptic plasticity rather than entrainment.

### Causal Role of Oscillations for Conscious Vision

In sum, both behavior (i.e., task performance) and neuronal oscillations can be affected by rhythmic sensory stimulation or rhythmic brain stimulation. Have these techniques been applied to oscillatory correlates of consciousness? If human brain oscillations can be controlled through entrainment approaches, oscillatory power and phase in specific frequencies become independent variables, allowing us to probe their causal role in conscious vision.

Using visual stimuli, Mathewson et al. (2010) found that detection performance depends on the latency of target presentation relative to a preceding rhythmic visual cue train. In fact, visual perception performance can oscillate across multiple alpha cycles following an alpha cue train (Mathewson et al., 2012; de Graaf et al., 2013). It seems likely that phase-reset/-locked neuronal alpha oscillations underlie such patterns of visual performance, as even a single sound can induce visual excitability fluctuations with alpha frequency (Romei et al., 2012).

In a pioneering study, Romei et al. (2010) showed that a burst of TMS pulses applied at 10 Hz directly affected whether or not a subsequent visual target was perceived. TMS pulses applied at different frequencies (5 or 20 Hz) had no such effect. Presenting visual targets at different latencies from a rhythmic alpha TMS burst also modulated target perception, suggesting that not only alpha power, but also alpha phase is causally relevant (Jaegle and Ro, 2014). Chanes et al. (2013) used TMS to entrain high-beta (30 Hz) or gamma (50 Hz) frequencies. They showed that neural activity was entrained only when these two specific frequencies were used, but not when the stimulation did not have a specific rhythm (used as control conditions). Depending on the frequency of stimulation, specific behavioral aspects of task performance were altered, such as perceptual sensitivity and response criterion.

The causal role of oscillatory activity in conscious vision has also been studied with tACS. Helfrich et al. (2014) suggest that tACS-entrained alpha phase is relevant for visual perception. Kanai et al. (2008) reported that with ambient light, it is possible to induce phosphenes with occipital tACS at beta frequency. In contrast, in darkness, phosphenes were more likely perceived with tACS at alpha frequency. In a recent study, a ‘square’ of two sets of diagonal light stimuli were presented in alternation (a ‘motion quartet’). In this bistable apparent motion stimulus, two lights could be perceived as moving back and forth horizontally, or vertically. TACS was applied at 40 Hz over both occipital cortices. The stimulation led to a relative decrease in horizontal motion perception, but only if the two hemispheres were stimulated with a 180 degrees phase difference (i.e., anti-phase) and not with 0 degrees phase difference (in-phase) (Strüber et al., 2014).

In sum, entrainment approaches allowing researchers to control the power or phase of oscillations at a particular frequency have indeed been applied to conscious vision paradigms. But at the same time, comparing these studies with the overview of oscillatory correlates makes clear that (1) many oscillatory correlates of consciousness remain to be tested causally using entrainment techniques, and (2) the reviewed entrainment studies have focused predominantly on local power and phase, while conscious perception might depend (also) on more complicated oscillatory mechanisms, such as widespread coherence. Therefore, it seems useful to quickly review some of the exciting recent developments in entrainment methodology, which may open up causal studies of oscillatory mechanisms of consciousness even further.

### Advanced Entrainment Approaches

Polanía et al. (2012) successfully manipulated oscillatory *coherence* between frontal and parietal cortex in a memory task, using tACS. Experimentally synchronizing oscillations in the theta (6 Hz) band (applying tACS over both regions with 0 degree phase difference) improved working memory performance, while experimentally desynchronizing oscillations (tACS over both regions with 180 degree phase difference) impaired performance. In another application, Alekseichuk et al. (2016) recently modulated *cross-frequency coupling*, showing that gamma bursts coinciding with theta-peaks improved working memory performance, while this effect was absent if gamma bursts coincided with theta-troughs. This experimental manipulation was achieved with tACS stimulation, with short bursts of gamma-signals superimposed on an ongoing theta-signal.

In principle, such sophisticated tACS entrainment approaches require only an appropriate electrode montage, and equipment that allows external control of electrical stimulators. A complex electrical waveform such as required for cross-frequency coupling modulation can ‘simply’ be programmed and fed into the stimulation devices. Also the presentation of stimuli in single or multiple modalities can be time-locked to one or multiple tACS waveforms, to for example consistently present certain inputs at certain phases. We recently discussed hardware and freely available software solutions to enable such experiments (ten Oever et al., 2016). While the examples discussed directly above did not relate to conscious vision, most of the oscillatory correlates of consciousness reviewed here could be causally studied with entrainment, using such available tools.

## Functional Roles of Oscillatory NCCs

Oscillatory mechanisms that covary with conscious experience are, by definition, NCC. Such empirical findings can be called ‘empirical NCCs’ (de Graaf and Sack, 2015). But it has been repeatedly noted that finding empirical NCCs is not the end goal. An empirical NCC can still fulfill different functional roles, which should be understood in order to move forward to understanding how the brain actually establishes conscious experiences (Miller, 2001, 2007; Noë and Thompson, 2004; Bachmann, 2009; Hohwy, 2009; Melloni and Singer, 2010; Aru et al., 2012; Kanai and Tsuchiya, 2012; Sergent and Naccache, 2012). In the context of oscillatory correlates of consciousness, this is exactly why entrainment approaches are so valuable; they allow us to go beyond correlation.

### Prerequisites, Substrates, Consequences

Several authors proposed different frameworks with possible roles that neural correlates, including oscillatory correlates, may play. What they appear to have in common, at least on a conceptual level, is that among the wealth of empirical neural correlates, only some reflect conscious experience itself.

Sergent and Naccache (2012) discuss the Global Workspace model (see also Baars, 1989; Dehaene and Naccache, 2001; Dehaene et al., 2006), postulating that many brain networks are continuously active, processing incoming information unconsciously. When top-down attention comes into play and leads to non-transient coherent activity throughout the brain, information can become conscious. In a first step, at low-level areas in the visual hierarchy (e.g., primary visual cortex) for about 200 ms after presentation of the stimulus, visual information is not yet conscious (‘upstream processing’). As the information spreads to higher-order areas (i.e., frontal lobes), in a second step we can reach ‘ignition’ of the global workspace. Ignition means that we will have conscious experience on the one hand, and several ‘downstream’ processes that result from conscious experience and its underlying neural signature on the other hand. These can be hard to distinguish.

In another framework, Ruhnau et al. (2014) suggest that the parameters of power and phase are useful to describe local excitability and consequent stimulus detection, but not sufficient to thoroughly explain conscious experience. In fact, they propose that other networks in the brain (connected to high-order areas, i.e., parietal and prefrontal) need to be pre-activated to open a so-called “window to consciousness” (*Win2Con*) and allow conscious perception. Local cortical excitability seems to be a “prerequisite” for conscious perception but does not reflect its neural process. General brain connectivity (from local to global level) seems to be required for visual consciousness, leading to conscious experience only when integration of relevant areas is achieved.

We and others (Aru et al., 2012; de Graaf et al., 2012; de Graaf and Sack, 2014) suggest that to define (and refine) correlates of consciousness it is useful to distinguish three core roles of an empirical NCC: neural *substrates*, neural *prerequisites*, and neural *consequences* of a conscious experience. ‘Substrates’ are the ‘actual’ NCC of interest, in the sense that the neural substrates of experience are directly causing, or are identical with, the phenomenal conscious experience. ‘Prerequisites’ are the neural events and mechanisms that are needed for neural substrates (and thus for a conscious experience) to arise. Consequences are in a sense less interesting, because they merely occur as a side-product of the neural prerequisites/substrates, however, meaningful in a cognitive/behavioral sense they may be. All the same, only a correct understanding, or even allocation, of empirical NCCs in light of these three different ‘roles’ can lead to a complete model of brain-experience relationships. Looking at the other examples of theoretical frameworks, it is easy to draw parallels. So we will continue to use our own terminology to refer to, for instance, ‘prerequisites’ rather than ‘upstream processes,’ even if similar conclusions could arise.

### Oscillatory Prerequisites, Substrates, and Consequences?

It might be useful to evaluate how this taxonomy maps onto oscillatory NCCs of conscious experience reviewed so far. This will not be exhaustive, to avoid repetition, but rather an exercise and illustration of the core concepts. For instance, it immediately becomes clear that many of the previously discussed empirical findings may fall in the ‘prerequisites’ category (Ergenoglu et al., 2004; Hanslmayr et al., 2007; Romei et al., 2008a; van Dijk et al., 2008; Busch et al., 2009; Lange et al., 2014). After all, any neuronal mechanism that occurs *prior* to a conscious experience can by definition not be a neural substrate or neural consequence of a conscious experience (de Graaf et al., 2012). In other words, beta, gamma, but most notably alpha power, phase, and coherence that occur before or at the moment of stimulus presentation, are either not required for conscious experience, or are prerequisites for it. They are empirical neural correlates, they can cause a conscious experience (later), but they cannot underlie the conscious experience itself (i.e., they are not substrates). This is because when the stimulus appears on a computer screen, there is not *immediately* a conscious experience of that stimulus. The visual information still needs to affect the retina, undergo rudimentary processing along several subcortical stations, reach primary visual cortex to be processed further, and only from that point onward could one reasonably start to wonder whether neural processing is or is not a substrate of a conscious experience (e.g., Silvanto et al., 2008).

On the one hand, one might argue that oscillatory phase at stimulus onset is reflective of oscillatory phase in the near-future. If one speculates that relevant visual processing occurs in primary visual cortex around 100 ms after stimulus onset (e.g., de Graaf et al., 2014), then alpha oscillations should actually be at the same phase when the information reaches the cortex, as was measured at stimulus onset. Thus, *indirectly*, peri-stimulus oscillatory correlates might still provide clues on neural substrates of consciousness. On the other hand, it is unclear at the moment to what extent the presentation of the stimulus itself changes ‘ongoing’ oscillations, for instance causing an oscillatory phase-reset. Such considerations make it all the more important that some studies try to bypass certain sensory processing stages, for instance by magnetically stimulating occipital cortex directly. It is thus non-trivial that similar alpha power/phase effects on conscious experience (phosphene perception) were found in these studies (Romei et al., 2008a; Dugué et al., 2011).

**FIGURE 4:**
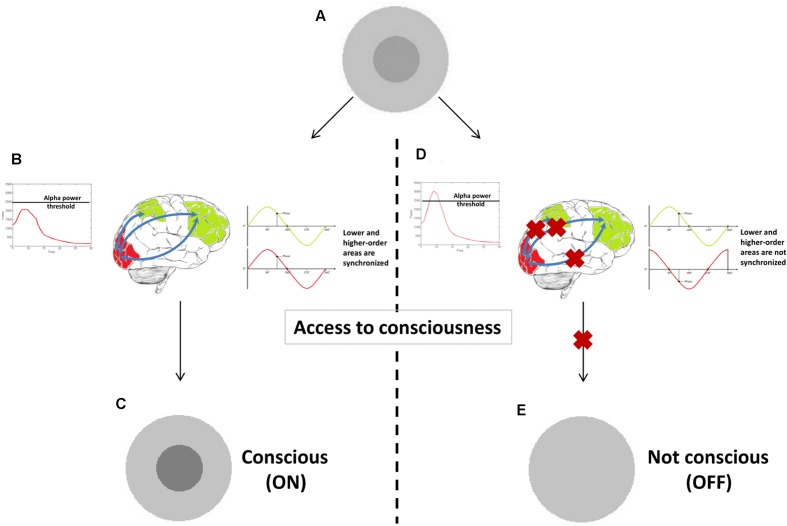
A proof-of-concept illustration of how different oscillatory correlates could constitute prerequisites versus substrates. When a stimulus is presented at sensory threshold **(A)**, it causes conscious experience **(C)** only if alpha power is sufficiently low (*prerequisite* in this example). Other oscillatory correlates (long-range synchronization in gamma or beta band in this example) can then arise **(B)**, underlying the experience itself (*substrates*). If one of these two oscillatory mechanisms is not present **(D)**, conscious experience is not achieved **(E)**.

In **Figure 4** a tentative model provides a *hypothetical* example of how *prerequisites* and *substrates* of consciousness may be related to different oscillatory correlates. We explained that a stimulus presented near sensory threshold may cause conscious experience depending on the brain state at the moment of its appearance. It might be that when power and phase of oscillatory activity fall under favorable circumstances (e.g., local alpha power in sensory – visual – areas has a momentary state below a particular threshold), they constitute (some of the) *prerequisites* necessary for a stimulus to become conscious. At this stage conscious experience is not yet achieved. Only when other mechanisms are engaged (e.g., long-range beta or gamma synchronization between low and high-order areas) conscious experience arises. The big challenge is to determine which of these additional processes are *substrates* of conscious vision.

Other empirical results presented here could potentially be reconsidered similarly. Gamma power, for example, was a long-standing candidate NCC. But there are recent findings that suggest that gamma oscillatory power is not, in the end, absolutely and always necessary nor sufficient for conscious experience (Luo et al., 2009). Formally speaking, that would mean gamma oscillatory power is not a universal prerequisite. But it could also be that it is required for some conscious experiences, such as coherent percepts that require binding of different visual features, and as such a prerequisite for specific experience and what one might call a precursor of conscious perception. Future studies should illuminate this issue, also clearly separating oscillatory gamma power from gamma-band coherence across regions.

The same could be said for beta-band oscillations. We did not cover many studies focusing on beta, but beta-band coherence still seems to be a candidate NCC. Beta-band responses to conscious perception seem to occur on a temporal scale that is consistent with conscious visual experiences, thus deserving further study. At the same time, one of the two main studies discussed that related beta-band oscillations to conscious perception actually employed the attentional blink paradigm (Gross et al., 2004), which leads us to arguably the largest confounder in NCC research: attention.

### Attention, Consciousness, Oscillations: Blurred Lines

Consciousness rarely seems to occur without attention, leading many researchers to argue that attention and consciousness are inextricably connected, if not the same process (Posner, 1994; Chun and Wolfe, 2000; O’Regan and Noe, 2001). Yet, others have argued that they are distinct phenomena, with distinct functions and neuronal mechanisms (Iwasaki, 1993; Lamme, 2003; Dehaene et al., 2006; Koch and Tsuchiya, 2007). Some recent neurophysiological evidence showed that a dissociation is not completely established (Chica and Bartolomeo, 2012), yet there are empirical demonstrations that reveal separated or even opposite effects of attention manipulations versus stimulus visibility (i.e., conscious perception) manipulations (van Boxtel et al., 2010; Watanabe et al., 2011).

The finer points of this ongoing discussion are beyond the scope of this review, but it is important to realize that many empirical correlates reviewed here can, in fact, also be interpreted as correlates of attention, rather than consciousness. Or at least, attention as a confounder can only rarely be ruled out. Many ON–OFF paradigms involve visual detection tasks, which could be said to either capture attentional efficacy, or conscious access. We saw that alpha power predicts conscious visual experience, but alpha power also indexes performance on explicit attention tasks (Thut et al., 2006). One example paradigm that demonstrates the entanglement of attention, conscious experience, and their relation to oscillations, is the attentional blink paradigm.

As we saw above, alpha oscillations play a prominent (yet not fully clear) role in attention and consciousness. In most attentional blink studies, the presentation rate for targets and distracters is approximately 10 Hz. Recent work has addressed the idea that the rhythmic stream of inputs in the attentional blink paradigm actually entrains alpha oscillations (Moratti et al., 2007). This phase-locking appears to result in visual stimulus presentation coinciding with troughs of EEG-measured parieto-occipital alpha oscillations (Hanslmayr et al., 2011). Moreover, pre-stimulus alpha phase at the onset of T1 predicts whether or not T2 will be detected (Zauner et al., 2012). These findings are in line with another study which suggests that, under strict temporal constraints, the processing of the pre-target distracter stream enhances phase locking of the alpha oscillation, which predicts lower T2 detection (Petro and Keil, 2015). Lastly, the notion that oscillatory entrainment is somehow involved in attentional blink suppression is supported by the fact that introducing temporal discontinuities in the RSVP stream around presentation of T1/T2 reduces the attentional blink effect (Martin et al., 2011).

## Conclusion

To chart the exact cascade of neurocognitive events leading from visual inputs to eventual button presses, with attention and a conscious experience somewhere along the way, is still an enormous challenge. It is clear that oscillatory mechanisms are part of this process, but even when focusing on the visual modality, there is no single oscillatory mechanism that emerges as the core candidate for conscious processing. The tools to tease apart the role of various reported oscillatory correlates of consciousness are still evolving, with sophisticated developments in tACS entrainment procedures as a recent methodological highlight. Different parameters of oscillatory activity, frequency, power, phase, and coherence, should be evaluated with these new tools, to eventually distinguish their roles and contributions.

## Author Contributions

All authors listed have made a substantial, direct and intellectual contribution to the work, and approved it for publication.

## Conflict of Interest Statement

The authors declare that the research was conducted in the absence of any commercial or financial relationships that could be construed as a potential conflict of interest. The reviewer JK and handling Editor declared their shared affiliation, and the handling Editor states that the process nevertheless met the standards of a fair and objective review.
